# Update on Progress in Selected Public Health Programs After the 2010 Earthquake and Cholera Epidemic — Haiti, 2014

**Published:** 2015-02-20

**Authors:** J. Wysler Domercant, Florence D. Guillaume, Barbara J. Marston, David W. Lowrance, Haiti Health Systems Recovery Team, Ministry of Health, Republic of Haiti

**Affiliations:** 1 Centers for Disease Control and Prevention, Haiti; 2 Ministry of Health, Haiti; 3 Division of Global Health Protection, Center for Global Health, CDC

On January 12, 2010, an earthquake devastated Haiti’s infrastructure, killing an estimated 230,000 persons and displacing more than 1.5 million (1). Ten months later, Haiti experienced the beginning of the largest cholera epidemic ever reported in a single country (2). Immediately after the earthquake and at the start of the cholera epidemic, health priorities in Haiti included improvement of surveillance and laboratory capacity for addressing public health threats in the general population and targeted surveillance and provision of improved water and sanitation in camps for internally displaced persons. As part of a multi-sector, post-earthquake response in collaboration with the Government of Haiti and others, CDC focused on supporting the recovery, expansion, or establishment of several key health programs (3). This update reports progress in selected health programs, services, and systems in Haiti as of the end of 2014.

## Human Immunodeficiency Virus Treatment

Although severely affected by the earthquake, human immunodeficiency virus (HIV) clinical services supported by the President’s Emergency Plan for AIDS Relief were restored quickly. For example, from October 1, 2008, to September 30, 2009, 44% (1,881 of 4,275) identified HIV-positive pregnant women received antiretroviral medicines to prevent HIV transmission to their children. This coverage fell to nearly 32% (1,115 of 3,563) the following year, which included the earthquake, but rebounded to 87% (4,783 of 5,502) (Figure) from October 2013 to September 2014. Similar recoveries occurred for other services, including antiretroviral therapy enrolment and screening of tuberculosis patients for HIV infection (4).

## Immunization Cold Storage Capacity

To support immunization programs in Haiti, there was a critical need to improve cold storage capacity at central, regional, and peripheral levels. Compared with the immediate post-earthquake period, cold storage capacity at the central level has increased from 17,000 L to 32,500 L, allowing for sufficient vaccine storage capacity until 2018. To date, regional-level storage capacity has increased by 212% since 2010; at the peripheral level, 86% of institutions have refrigerators, with plans to reach 100% coverage of institutions with solar refrigerators over the next 3 years (5). Combined with other efforts, the improvements in cold chain capacity have facilitated the scale-up of measles-rubella vaccination (Figure) and the introduction of two vaccines into the routine immunization schedule: a pentavalent vaccine that protects children against five diseases (diphtheria, tetanus, pertussis [whooping cough], hepatitis B, and *Haemophilus influenzae* type b [Hib] disease) with one shot (2012) and rotavirus vaccine (2014).

## Lymphatic Filariasis Elimination

The elimination of lymphatic filariasis in Haiti was at risk at the time of the earthquake because of a lack of campaigns to administer drugs to treat the disease in Port-au-Prince. Subsequently, with CDC support, Haiti achieved 93% national coverage for lymphatic filariasis mass drug administration (MDA) among at-risk persons, with approximately 8 million persons treated nationally, including 2.3 million in Port-au-Prince (4) (Figure). In all, 58% of communes in Haiti have completed five or more rounds of MDA. Currently, 13 evaluation units in the regions of Centre, Nippes, Nord, Nord Est, Nord Ouest, and Sud Est are eligible and scheduled for Transmission Assessment Surveys in 2015 to determine if MDA can be stopped (6). For the remaining regions, MDA will continue as needed, with metropolitan Port-au-Prince set to receive its fourth and fifth rounds of MDA in 2015 and 2016, respectively.

## Malaria Testing

Elimination goals for malaria were predicated on a targeted “test and treat” approach; there was a need to expand diagnostics to move from treatment of clinically diagnosed malaria to the treatment of laboratory-confirmed cases. To reach this objective, in 2010 the malaria program approved a national policy for use of rapid diagnostic tests. As of March 2014, approximately 900 staff members from 214 institutions received training on new case management guidelines and approximately 300,000 rapid diagnostic tests were conducted, bringing Haiti closer to the malaria pre-elimination stage (7).

## Cholera Response

Efforts to respond to and control the cholera epidemic ultimately contributed to a large decline from 352,033 cases in 2011 to 15,063 through October 2014, with annual case fatality rates consistently below the World Health Organization target of 1% since 2011 (8). Extremely weak water and sanitation infrastructure and services, which contributed to the rapid spread of cholera, have been addressed in rural areas by the hiring and training of more than 250 rural water and sanitation technicians. Of the more than 500 rural community-based piped water systems identified in a national survey, a chlorination program has been implemented in 107 (9). In Port-au-Prince, results from a CDC evaluation of the quality of water sold by private vendors indicated that the water was usually pathogen-free at the point of sale.

## Rabies Control

Haiti has the highest incidence of human rabies in the Western Hemisphere, and a lack of rabies surveillance data has limited control efforts. An initiative of the Haitian Ministry of Health and CDC begun in July 2013 is addressing that deficiency. From July 2013 to June 2014, CDC and Haitian Ministry of Health staff members investigated 323 possible cases of canine rabies, with 27% found positive or probable. For the metropolitan Port-au-Prince area, this represented a 14-fold increase in the number of cases investigated compared with the same period in 2012, when only 23 cases were investigated (10).

## Laboratory Diagnosis of Tuberculosis

Laboratory diagnostic capacity for tuberculosis has expanded from no site with fluorescent microscopy and one site with nucleic acid amplification equipment in 2010 to 25 sites with fluorescent microscopy and 12 sites with nucleic acid amplification capacity in 2014. This has contributed to improved detection and notification of cases of active tuberculosis; the World Health Organization estimates that the rate of detection of active tuberculosis cases in Haiti improved from 62% in 2010 (11) to 80% in 2013 (12).

## Sentinel Surveillance for Notifiable Diseases

The national sentinel surveillance system (13) for notifiable diseases has expanded from 51 sites soon after the earthquake to 153 currently. The system has facilitated identification and investigations of immediately notifiable diseases, including vaccine-preventable diseases such as measles, rubella, and diphtheria. In addition, a system for enhanced laboratory-based surveillance strengthens analysis of predominant etiologies of acute febrile, respiratory, and diarrheal diseases, and meningitis.

## Field Epidemiology Training

Since 2011, the Haitian field epidemiology training program has graduated 154 basic-level residents, 58 intermediate-level residents, and five advanced-level residents. These graduates have supported disease control efforts for a number of outbreaks, including cholera, dengue, and chikungunya, and have developed a national Ebola preparedness plan (14).

## New Challenges and Priorities

Progress in Haiti remains fragile. For example, although there have been significant accomplishments and improvements in both rural and urban settings, there remains a great shortfall in the resources required for water and sanitation infrastructure and services to eliminate cholera in Haiti, as outlined in the country’s 10-year national plan (15). There has been slow and limited progress in restoring the physical health infrastructure. For many programs, the financial resources that were made available following the earthquake were needed simply to maintain key programs, with a focus on human resources and commodities; funding sources for ongoing, essential public health programs remain uncertain.

As the focus of the response has naturally shifted from addressing short-term crises to long-term needs, new challenges and priorities have emerged. However, the link between immediate problems (e.g., cholera) and structural issues (e.g., weak water and sanitation systems) is clear, and the two need to be addressed in tandem. Failure to consider the long-term context in the immediate aftermath of an emergency (e.g., local human capacity to sustain a program) might jeopardize hard-won public health gains over time. Nonetheless, Haiti has seen substantial progress in key health indicators in just 5 years and might serve as a model for other resource-limited countries recovering from natural or manmade disasters, including countries prioritized under the global health security agenda and those recovering from the West African Ebola epidemic (16,17). Additional information related to activities supported by CDC in Haiti is available at http://www.cdc.gov/globalhealth/countries/haiti and via Twitter at @CDCHaiti.

## Figures and Tables

**FIGURE:**
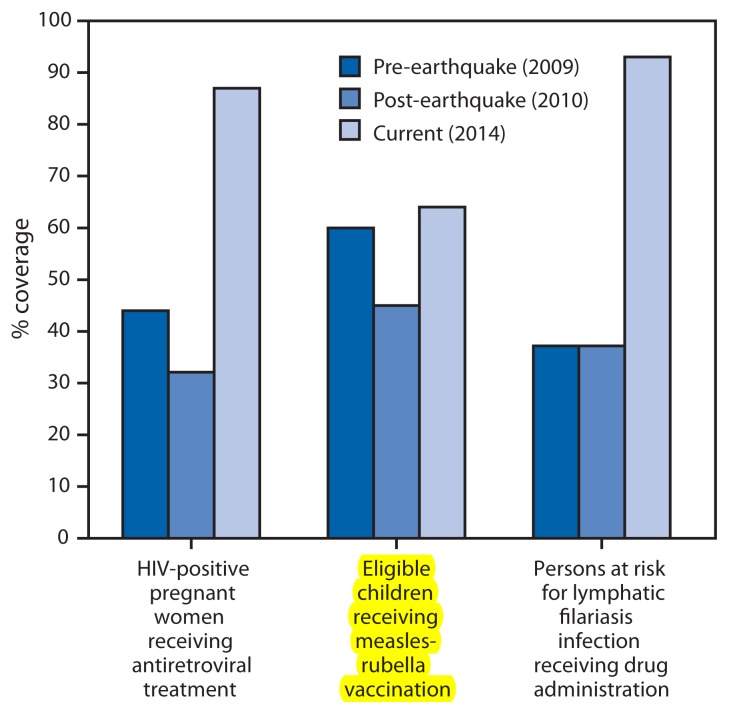
Selected measures of public health program progress — Haiti, 2009–2014
